# Deep Convolutional Neural Network for Mapping Smallholder Agriculture Using High Spatial Resolution Satellite Image

**DOI:** 10.3390/s19102398

**Published:** 2019-05-25

**Authors:** Bin Xie, Hankui K. Zhang, Jie Xue

**Affiliations:** 1 Institute of Remote Sensing and Earth Sciences, Hangzhou Normal University, Hangzhou 311121, China; xiebin@hznu.edu.cn; 2 Geospatial Sciences Center of Excellence, South Dakota State University, Brookings, SD 57007, USA; 3 Department of Geography and Resource Management, The Chinese University of Hong Kong, Shatin, Hong Kong, China; jiexue@link.cuhk.edu.hk

**Keywords:** deep convolutional neural network (CNN), high spatial resolution, GaoFen-1, smallholder agriculture

## Abstract

In classification of satellite images acquired over smallholder agricultural landscape with complex spectral profiles of various crop types, exploring image spatial information is important. The deep convolutional neural network (CNN), originally designed for natural image recognition in the computer vision field, can automatically explore high level spatial information and thus is promising for such tasks. This study tried to evaluate different CNN structures for classification of four smallholder agricultural landscapes in Heilongjiang, China using pan-sharpened 2 m GaoFen-1 (meaning high resolution in Chinese) satellite images. CNN with three pooling strategies: without pooling, with max pooling and with average pooling, were evaluated and compared with random forest. Two different numbers (~70,000 and ~290,000) of CNN learnable parameters were examined for each pooling strategy. The training and testing samples were systematically sampled from reference land cover maps to ensure sample distribution proportional to the reference land cover occurrence and included 60,000–400,000 pixels to ensure effective training. Testing sample classification results in the four study areas showed that the best pooling strategy was the average pooling CNN and that the CNN significantly outperformed random forest (2.4–3.3% higher overall accuracy and 0.05–0.24 higher kappa coefficient). Visual examination of CNN classification maps showed that CNN can discriminate better the spectrally similar crop types by effectively exploring spatial information. CNN was still significantly outperformed random forest using training samples that were evenly distributed among classes. Furthermore, future research to improve CNN performance was discussed.

## 1. Introduction

Land cover plays an important role in human-environment interactions [1,2,3,4] including nature resource management, precision agriculture, ecosystem service modelling, climate change and urban planning, and has been recognized as an essential climate variable by the Global Climate Observing System (GCOS). Land cover mapping was undertaken since the first acquisition of satellite images [5]. Over the last several decades, land cover mapping has evolved from low to high spatial resolution [6,7], from local to global scale [8,9] and from single to time series image utilization [10,11]. This is attributed to the advancements in open data policy [12], analysis ready data [13], and machine learning and computation facilities [4].

The state-of-the-art of remote sensing image classification is to use non-parametric supervised classifiers, e.g., support vector machine [14] and random forest [15], trained with collected land cover samples. However, these machine learning classifiers need hand-engineered features to achieve good performance. Recently, deep convolutional neural network (CNN), due to its capability to extract features from raw data (end-to-end training), has significant advantage [16] in natural image scene classification in the computer vision field and has been proven in land cover mapping [17,18,19,20] (Table 1). The deep CNN originates from deep neural network, which has been in conception for decades [21] but only trainable and applicable after recent breakthrough in understanding of deep neural network [22]. The deep CNN has better representative capability as it usually consists of several to hundreds of feature layers linked by millions of learnable parameters to map the input predictors to the output class labels [16]. Convolution in CNN is usually applied on an image patch rather than a single pixel so that the high level spatial features are extractable. A convolution layer is usually followed by pooling operation, which combines several nearby features to a unique feature, to reduce noise, improve CNN efficiency [23], and keep scale and translational invariance features [24]. The popular pooling operation is max or average pooling [23], i.e., taking the max or average value of the nearby features as the pooling output.

Deep CNN has been used for detecting anomalies [25] and weeds [26] in agricultural field and for crop specie recognition [27] among many other agricultural applications [28]. A few studies have used deep CNN for cropland classification with median [29,30] and high [31,32] spatial resolution satellite images. The agricultural landscape is known to be difficult to classify reliably [33,34,35] especially smallholder crop areas [36,37,38] with field size smaller than 5 ha [36]. The smallholder agricultural landscape is mainly distributed in developing countries including China [39,40]. To map these heterogonous landscapes, effectively exploring spatial information is the key as many different crop types with similar spectral pattern coexist. The CNN is good at exploring spatial information, but the CNN structure parameters need to be carefully tuned to maximize its performance. This is because the optimal CNN structure may depend on the satellite image characteristics, input image patch size and training sample characteristic, e.g., Table 1 lists the CNN structure variety in the literature for remote sensing image classification. 

The objectives of this study are to (1) apply the deep CNN to classify high spatial resolution images acquired over smallholder agricultural landscapes and (2) to compare CNN with the established random forest classifier. This study uses pan-sharpened GaoFen-1 images with 2 m spatial resolution over four areas in Heilongjiang, China with smallholder agriculture. The reference land cover maps are interpreted from 2 m GaoFen-1 images and systematically sampled for training and testing. The CNN structure parameters are tuned with different pooling strategies and with different complexities. The classification results are evaluated using conventional confusion matrix derived from the testing samples and visual comparison of the classification maps.

## 2. Data

### 2.1. Study Areas

**Figure 1:**
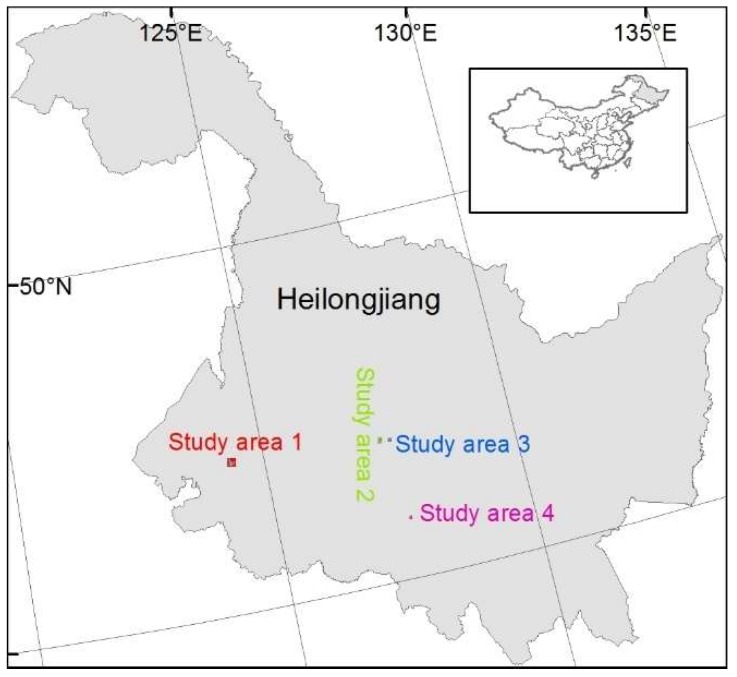
Four study area locations in Heilongjiang province, China.

The study areas are in Heilongjiang province, which is located in the northeast agricultural area of China (Figure 1). The agricultural land accounts for 83.5% of the 473,000 km^2^ province land area. Four study areas including Dangnai village in Dorbod Mongol autonomous county (study area 1), Jufu village in Qing’an county (study area 2), Jiuwang village in Qing’an county (study area 3) and Changhe village in Bin county (study area 4) were chosen. The study area 1 is mainly covered by wetland and maize, study areas 2 and 3 by rice, and study area 4 by maize.

### 2.2. GaoFen-1 Data

The panchromatic/multispectral data from the China GaoFen-1 satellite were used in this study. The GaoFen-1 was launched in 2013 and provides 10-bit radiometric resolution panchromatic and multispectral data with 2 and 8 m spatial resolutions, respectively (Table 2). The detailed characteristics of GaoFen-1 data are summarized in Table 2. 

**Figure 2:**
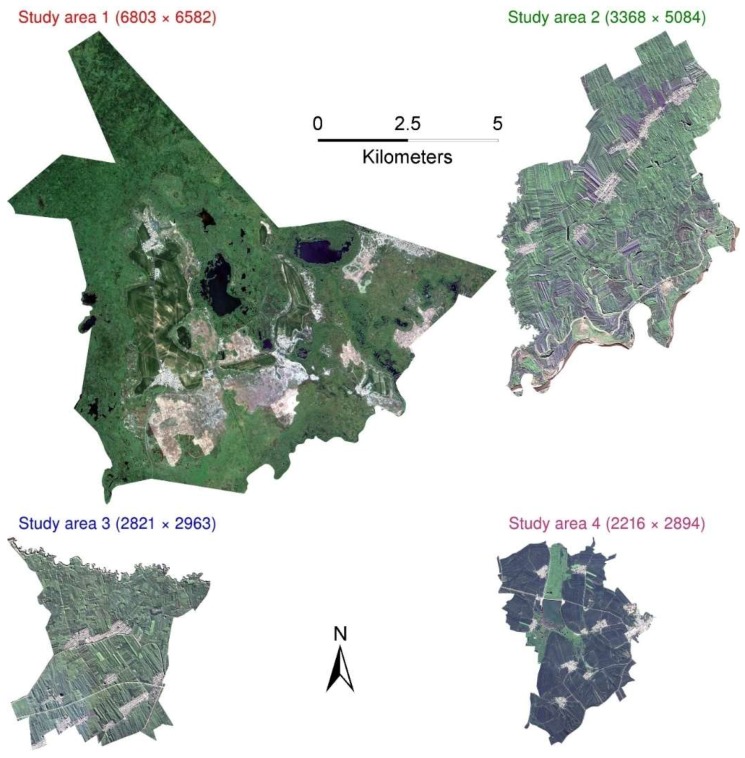
Four study area (locations shown in Figure 1) GaoFen-1 true color images with 6803 × 6582, 3368 × 5084, 2821 × 2963 and 2216 × 2894 2 m pixels. Their sizes are proportional to their areas.

In this study, four GaoFen-1 panchromatic/multispectral images (Figure 2) over the four study areas were collected on 22 August, 13 August, 13 August and 17 August, 2016, respectively. All the 8 m four band multispectral images were pan-sharpened to 2 m using the well-established Gram–Schmidt method which is known to have moderate computational load and good spatial sharpening capability [65,66]. The images were mainly acquired in August considering that the harvest time of the main crops (wheat, rice, maize, etc.) in Northeast China is in September and October. They were subsequently cut according to the village administrative boundaries of the four study areas (Figure 2).

### 2.3. Reference Land Cover Maps for Training and Testing

**Figure 3:**
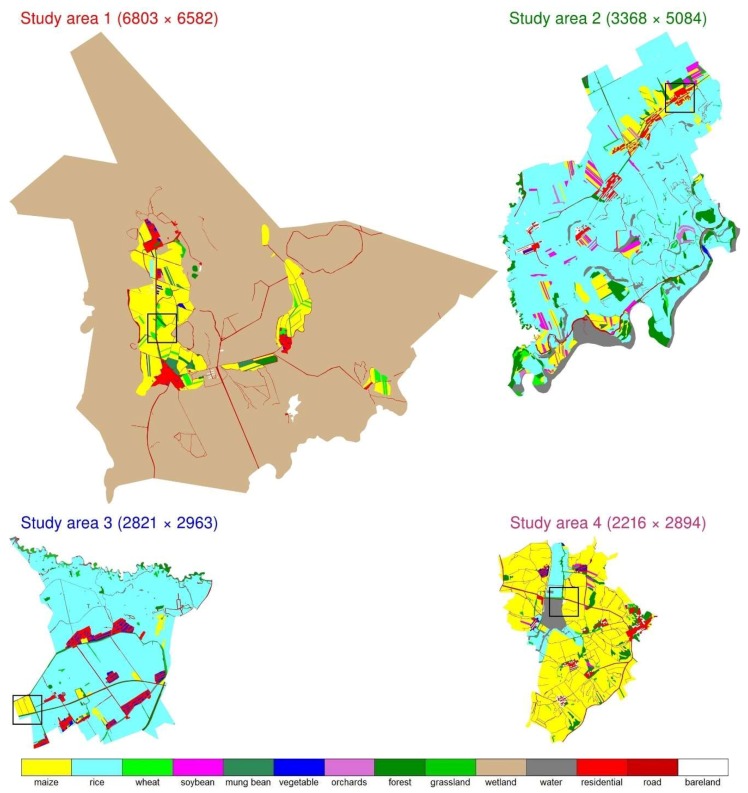
Four study area reference land cover maps (see Figure 2 caption for detailed information). The box covering 400 × 400 2 m pixels in each study area is examined in detail in the results section.

The reference land cover maps (Figure 3) were derived by visual interpretation of the 2 m pan-sharpened GaoFen-1 images with interpreter’s field survey knowledge [67]. For each GaoFen-1 image, three sample plots each with 200 × 200 m pixels were randomly selected and visited for land cover type identification by the interpreter in order to gain the necessary knowledge on the GaoFen-1 image characteristics of different land cover types. The entire study area image was then manually interpreted based on the knowledge. There are 14 land cover types including maize, rice, wheat, soybean, mung bean, vegetable, orchards, forest, grassland, water, wetland, road, residential and bare land. The small patch crop landscapes are evident in the reference landscapes (Figure 3). 

The training and testing samples were systematically collected from the reference land cover maps to make sure the proportional distribution among the classes related to the proportion that they occur in reality. Keeping class proportions in training samples has been shown with more reasonable classification accuracies [10,68]. The training and testing pixels cannot be very close to avoid that the spatial correlation may boost the testing data classification accuracy. They cannot be very far away to ensure enough training samples for the CNN model. Consequently, the reference land cover maps were sampled every five pixels in column and row directions. The sampled pixels in the pool were randomly and equally divided into training and testing samples. The training and testing sample numbers are shown in Table 3.

## 3. Methods

### 3.1. CNN Overview

**Figure 4:**
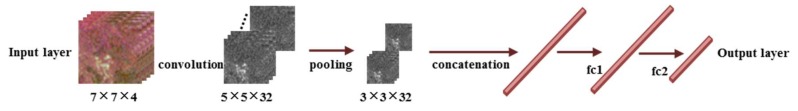
A typical CNN structure. The figure is adapted from Liu et al. [52].

CNN usually consists of multiple (several to more than one hundred) stacked layers each containing a certain number of features [16] (Figure 4) derived from the previous layer features by nonlinear transformation. Using these feature layers, the CNN is to convert the input *K* × *K* × *D* (*K* being the spatial neighbor size and *D* being the spectral band number, i.e., *D* = 4 in this study) image patch to one single class label of the center pixel. Each layer feature is derived from the previous layer features through mathematical transformation, which nominally contains a linear operation with learnable weight and bias parameters and a fixed non-linear activation function. The convolution layer uses convolution kernel (the kernel values are the learnable weights) to extract information from a small region’s neighbor pixels and contain many kernels to extract different feature information. The last convolution layer is usually followed by fully connected layers to extract more abstract features. These final, fully connected layer features are fed into a softmax function for classification. In such a way, the loss function can be defined using training samples with known land cover labels and the CNN training is to find the optimal learnable parameters to minimize the loss function. In this study, the cross-entropy loss function is used:

(1) $$loss\;=-\overset{C}{\underset{i=1}{\sum }}\left\{\left(y==i\right)*\log\frac{e^{a_{i}}}{\sum_{i=1}^{C}e^{a_{i}}}\right\},$$

where *C* is the total number of classes in the classification legend indexed by *i*, *y* is the ground truth label, *a_i_* is the *i*th feature value of the final fully connected layer with *C* features. This loss function is minimized using a mini-batch gradient descent method where each gradient descent iteration only uses a small portion of the training samples to prevent overfitting and to save computation load [69].

The convolution is formulated as below,

(2) $$v_{l,t}^{xy}=f\left(b_{lt}+\underset{k}{\sum }\overset{H_{l,t}-1}{\underset{p=0}{\sum }}\overset{W_{l,t}-1}{\underset{q=0}{\sum }}\omega_{ltk}^{pq}v_{l-1,k}^{\left(x+p\right)\left(y+q\right)}\right),$$

where $v_{l,t}^{xy}$ stands for the output at position (*x*, *y*) of the *t*th feature map at the *l*th layer, *f* denotes the non-linear activation function, $b_{lt}$ refers to the bias term, and *k* indexes over the set of feature maps of the (*l*-1)th layer, *H_l,t_* and *W_l,t_* are the height and width of *t*th kernel of *l*th layer and are indexed by *p* and *q*, respectively, $\omega_{ltk}^{pq}$ is the convolution kernel value at position (*p*, *q*) of the *t*th feature map in the *l*th layer connected to the *k*th feature map in the (*l*-1)th layer. The recently developed rectified linear units (ReLU) [70] is used as *f* in this study:

(3) f(x)=max(0,x).

For the edge pixels in the input image patch, the convolution is usually conducted by enlarging the input images with zero values (i.e., zero padding). Convolutions with zero padding can keep the input feature map size unchanged and lose little information.

**Figure 5:**
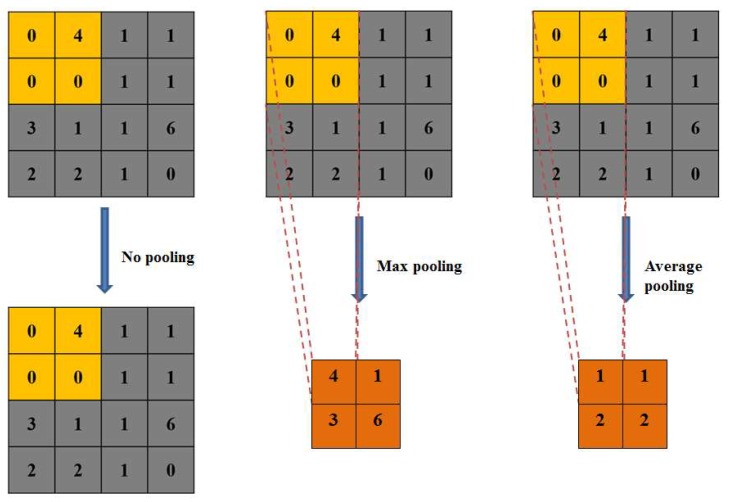
Illustration of no pooling (**left**), max pooling (**middle**) and average pooling (**right**) operations with 2 × 2 size and stride.

In the CNN used for object recognition [71], pooling is applied after convolution to derive more abstract and scale invariant features [24], and to reduce noise and avoid overfitting [23]. The pooling can resize the input feature maps spatially. For example, a commonly used max pooling with 2 × 2 size and 2 × 2 stride will go through the input feature map spatially along both width and height directions and take the maximum value of the 2 × 2 features in the input feature map Figure 5(middle). This will only keep a quarter of the input features and discard 75% of them.

### 3.2. CNN Structure Parameter Tuning

The input patch image size was set to 7 × 7 (*K* = 7) as many of the previous studies (Table 1) used such window to balance the computation efficacy and the classification accuracy. Moreover, larger window size may overly smooth the classification results since the classification is implemented on a sliding window basis. The convolution kernel size was set as 3 × 3 and the pooling size and stride size were set as 2 × 2 following previous studies [16]. 

Six CNN structures were designed using two different numbers of learnable parameters each with three different pooling strategies. The three different pooling strategies were (Figure 5): (1) no pooling was used and the feature map size was not reduced after convolution, for example, the input 7 × 7 image will become a 7 × 7 feature map no matter how many layers of 3 × 3 convolution were applied (Table 4; Figure 5(left)); (2) max pooling was used and feature map size was reduced after convolution and pooling, for example, the input 7 × 7 image will result in a 4 × 4 feature map after a 3 × 3 convolution and a 2 × 2 stride pooling and will result in a 1 × 1 feature map after three 3 × 3 convolution layers each followed by a 2 × 2 stride pooling (Table 4; Figure 5(middle)); and (3) same as (2) but using average pooling (Table 4; Figure 5(right)). Five layer CNN consisting of three convolution layers and two fully connected layers were used in this study. This is because pooling strategies (2) and (3) can have a maximum of three convolution layers (i.e., three convolution layers with 2 × 2 stride pooling will lead to a 1 × 1 feature map) and the convolution layers are usually followed by two fully connected layers.

Two CNN settings with different numbers of learnable parameters (~70,000 and ~290,000 in Table 4) representing different complexity were used to examine the CNN structure complexity effect. Each setting has very similar number of learnable parameters (i.e., similar complexity) so that pooling strategies in each set were fairly compared.

### 3.3. CNN Training Parameterization

In this study, all the CNNs were trained using the optimal hyperparameters for fair comparison. Following the convention, the input patch reflectance was normalized for each band by being subtracted by mean and divided by standard deviation. The CNN model was regularized using the weight decay method with coefficient of 0.001. The mini-batch size was set as 256. The learning rate started from 0.1 and decreased by 10 times when the validation sample accuracy stopped improving. The validation sample accuracy was calculated and compared every 100 mini-batch gradient descent iterations to check accuracy improvement. In the whole training process, decreasing the learning rate three times can achieve maximum accuracy (i.e., the learning rate changes from 0.1, 0.01, 0.001 to 0.0001). Following He et al. [72], the validation samples occupied 4% of the training samples (Table 3) for each study area and were randomly selected from them. The start learning rate was different from the recommended value of 0.01 in Krizhevsky et al. [16] and He et al. [72] because Ioffe and Szegedy [73] suggested that increasing the start learning rate can get better accuracy. We tested our CNN models and found that the 0.1 start learning rate did give slightly better accuracy without gradient explosion. Such high starting learning rate can be used because the batch normalization regularization technique [73] was adopted to normalize the features generated by each layer. The CNN was implemented using Tensorflow.

### 3.4. Classification Result Evaluation

The random forest classifier [15] was used as a benchmark for comparison. Random forest was run using the same 7 × 7 image patch as input. The random forest was implemented using the R software RANDOMFOREST package (http://www.r-project.org/). The default random forest parameter settings were used (e.g., 500 trees, the number of features used in each split is the squared root of the total number of features, and the number of samples in each tree is 63.2% of total without replacement).

The accuracy of the generated land cover map was validated both quantitatively and visually. The quantitative indices were only applied to the testing samples shown in Table 3 to avoid the accuracy boosting due to training and testing sample spatial correlation. Four quantitative indices, i.e., the overall accuracy, producer’s accuracy, user’s accuracy and kappa coefficient were used to evaluate the accuracy of classification of the six CNN structures and the random forest model. In addition, all the study areas were classified, and the land cover classification map was visually assessed by comparing with the true color GaoFen-1 image and the reference land cover map.

Although the imbalanced training samples are on purpose used so that they are proportional to the ground truth class occurrence [10,68], we also compared the random forest and the optimal CNN structure using the balanced training dataset. This is to avoid that the rare classes that are difficult to classify may bias the random forest and CNN comparison. For all the classes with more than 4000 samples, 2000 samples were randomly selected as training and 2000 samples (different from training) as testing. The overall accuracy, producer’s accuracy, user’s accuracy and kappa coefficient of the testing samples were tabulated and compared. 

## 4. Results

### 4.1. CNN Structure Parameter Tuning

**Figure 6:**
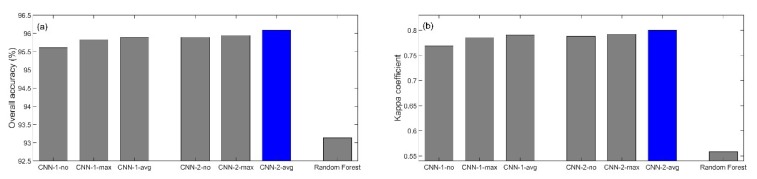

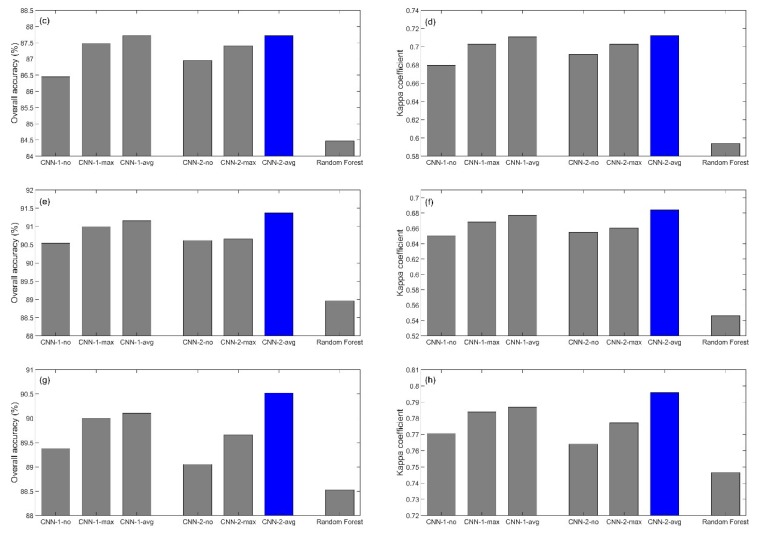
(**a**) and (**b**) show the six CNN model and random forest overall accuracies (**a**) and kappa coefficients (**b**) of the study area 1 testing sample classification. (**c**) and (**d**) show the same for study area 2 testing sample classification, (**e**) and (**f**) for study area 3 testing sample classification, and (**g**) and (**h**) for study area 4 testing sample classification. CNN1 and CNN2 indicate the first and second CNN structure settings with ~70,000 and ~290,000 learnable parameters, respectively. -no, -max and -avg indicate no, max and average pooling, respectively. The blue color is used to indicate the best performance CNN model among the six models.

Figure 6 shows, for each study area, the testing sample classification overall accuracies (left column) and kappa coefficients (right column) for the six CNN models and random forest. In Figure 6, the blue color is used to indicate which CNN model among the six gets the best performance so that CNN2-Avg always obtains the best scores. This value is directly compared to the performances obtained by random forest algorithm. The rank of three pooling strategies in the order of increasing performance is no pooling, max pooling and average pooling for all the study area experiments and for both CNN settings with significantly different numbers of learnable parameters. The accuracy advantage of the CNN structure with average pooling over the equivalent without pooling is moderate (0.2–1.5% higher overall accuracy and 0.01–0.04 higher kappa coefficient). This may be because the input image patch size is much smaller and there is not much image noise that pooling can suppress. Previous studies showed that either max or average pooling may perform best depending on the data feature distribution [23], while results in this study revealed that the GaoFen-1 data feature distribution is more favorable to average pooling strategy. All the CNN overall accuracies are greater than 85% indicating good classification performance.

For each CNN setting, the three pooling operations were on purpose designed to have very similar numbers of learnable parameters so that the three pooling operations were fairly compared. For the best pooling operation (i.e., the average pooling), the CNN model with ~290,000 learnable parameters (CNN2-Avg) performed slightly better than the light model with ~70,000 learnable parameters (CNN1-Avg). This is reasonable considering that more learnable parameters give more power to CNN representation. However, the more than four times learnable parameters only give <0.5% and <1% improvement indicating that the CNN structure used in this study has marginal improvement space without more complicated CNN techniques (e.g., skip connections).

### 4.2. CNN and Random Forest Classification Accuracy Comparison

The CNN2-Avg has 2.4–3.3% higher overall accuracy and 0.05–0.24 higher kappa coefficient (Figure 6) than the random forest using the same 7 × 7 input image patch indicting the more powerful capability of CNN to explore high level spatial structural information. The kappa coefficient improvement of the CNN model over the random forest model is much larger than the overall accuracy improvement because the user’s and producer’s accuracies are better balanced in the CNN model classification. Table 5 shows the user’s and producer’s accuracies for the four study area testing samples. For example, the rare class wheat in Table 5a has more than 60% user’s and producer’s accuracy difference for random forest and has only <13% difference for CNN classification. Other such rare class examples include rice, mung bean and bare land in Table 5a, wheat, orchards and road in Table 5b, maize, vegetable and road in Table 5c, and wheat, soybean and residential in Table 5d. Some user’s accuracy is NaN (Not a Number, indicating no value) as the total number of pixels classified as such class is zero and is used as dividend for user’s accuracy calculation. Note that the producer’s accuracy cannot be NaN as there is always some ground truth samples in the testing samples.

### 4.3. CNN and Random Forest Land Cover Map Comparison

**Figure 7:**
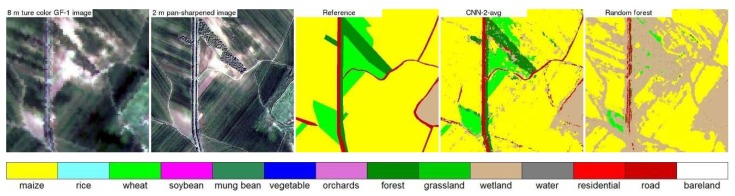
Study area 1 example 8 m GaoFen-1 true color image (the 400 × 400 2 m pixel box in Figure 3), 2 m pan-sharpened image, and three 2 m land cover maps (the reference land cover map, the land cover map generated by CNN model with average pooling and ~290,000 learnable parameters, and the land cover map generated by the random forest model). The two true color images are displayed using the same stretch.

**Figure 8:**
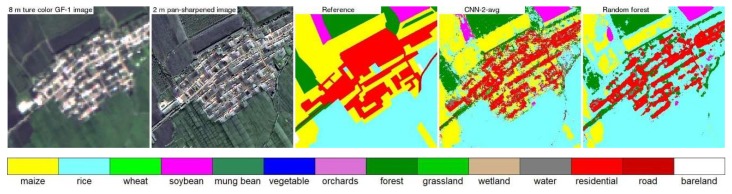
Same as Figure 7 but for the example 400 × 400 pixels in the box area in study area 2 (Figure 3).

**Figure 9:**
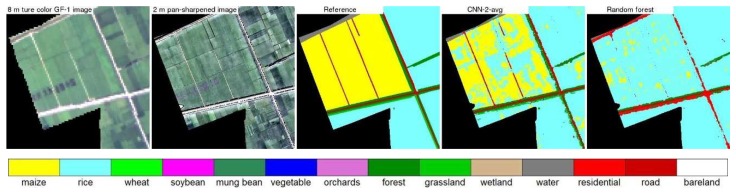
Same as Figure 7 but for the example 400 × 400 pixels in the box area in study area 3 (Figure 3).

**Figure 10:**
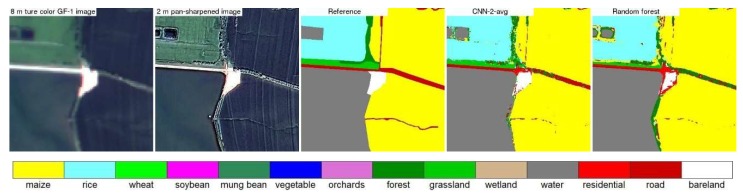
Same as Figure 7 but for the example 400 × 400 pixels in the box area in study area 4 (Figure 3).

Figure 7, Figure 8, Figure 9 and Figure 10 show the 8 m GaoFen-1 true color image, 2 m pan-sharpened image, reference land cover map, two land cover maps produced by the best CNN structure (CNN2 with average pooling) and the random forest models, for the four 400 × 400 2 m pixel example areas shown in Figure 3. The pan-sharpened images have more spatial details which are favorable for small crop field classification. The random forest classification maps are inferior to (i.e., less resembling to the reference land cover maps than) the CNN classification maps. This is because the end-to-end CNN training process can extract spatial information which is helpful to classify the spectrally similar classes, such as the maize and grassland classes in Figure 7, rice and wheat classes in Figure 8, rice and maize classes in Figure 9, and road and bare land classes in Figure 10. Due to the same reason, the linear features (e.g., roads and roadside forests in Figure 7, Figure 9 and Figure 10) are more evident in the CNN classification maps. 

### 4.4. CNN and Random Forest Accuracy Comparison for Balanced Training Data

All the previous results were derived from the training samples which are proportional to the land cover occurrence in the reference land cover maps. To examine the training sample balance effect, in this section, the CNN and random forest were trained using the sample data balanced among different classes, i.e., same number of training samples for all the classes. Table 6 lists the balanced testing dataset classification accuracies for the four study areas. The random forest is still inferior to the CNN model by 3.8–9.1% overall accuracy and 0.04–0.10 kappa coefficients. However, the overall accuracy of CNN model is only 78–85% that is lower than the imbalanced training dataset (Table 5). This is because overall accuracy may be boosted for imbalanced dataset by simply classifying most pixels as the majority classes. Similarly, the user’s and producer’s accuracies for CNN are more balanced than that for random forest. 

## 5. Discussion

CNN has been used for land cover classifications using remote sensing data, in particular hyperspectral data [42,43,48,49,64]. In this study, CNN was evaluated for mapping smallholder agriculture considering the unique spatial pattern of the smallholder agricultural landscapes. The CNN structures with three different pooling strategies were carefully designed with very similar numbers of learnable parameters and they were trained using the same optimal hyperparameters. The classification results showed that the pooling is still necessary even for small input image patch (i.e., 7 × 7 input image patch) because the small patch size input image still contains a moderate level of irrelevant information that the pooling can suppress. The average pooling performed better than max pooling because the max pooling is more suitable for separation of features that are very sparse [23] which is not the case for a small 7 × 7 input image patch. 

In this study, the simple Gram–Schmidt method was used to pan-sharpen the 8 m multispectral images. Its adoption can be considered a reasonable trade-off between performance and easy-to-use. The Gram–Schmidt method, in fact, has performance that can be considered acceptable, even though it is not an up-to-date method and other pansharpening methods produce better scores [74,75,76,77]. Anyway, its adoption presents undeniable advantages in terms of easy-to-use because Gram–Schmidt is included in commercial software. 

The CNN classifications have better accuracy than the random forest classifications as the CNN can explore the high level spatial information. However, there is still some pepper and salt effect in the CNN land cover classification maps. They occur mostly in the heterogeneous area, e.g., the residential and maize mixed area in Figure 8 and the roads and roadside forests mixed area in Figure 10. This is because in such heterogeneous area with two land cover classes distributed in a checkerboard pattern, all pixels may have very similar spatial context whatever the pixel class is. To handle such issue, Zhang et al. [56] and Pan and Zhao [78] have tried to fuse CNN and Markov based classification results at the decision level. Other options to handle this may use multi-scale CNN [44,79], object based CNN [80,81] and fully convolutional network [82,83]. 

The CNN had better performance than random forest for both imbalanced and balanced training datasets. In the imbalanced dataset, the classes with rare samples are difficult to classify and random forest easily misclassified these classes. The CNN performed much better for classes with rare samples and has a very high kappa coefficient improvement compared to the random forest. Furthermore, in this study the CNN loss function is defined as the average loss function of all the training samples. Training sample loss function can be easily weighted based on their classes to better classify specific classes. This may be useful when user’s accuracy is more or less important than the producer’s accuracy, for example, in cloud detection, omission error has a more severe effect on time series application than commission error [84]. 

## 6. Conclusions

This study evaluated CNN for remote sensing image classification of smallholder agricultural landscape using pan-sharpened GaoFen-1 satellite data with 2 m spatial resolution. Four study areas in Heilongjiang province were selected with reference land cover maps generated by manual interpretation. Pixels were systematically sampled from the reference land cover maps and evenly split for training and testing. Six CNN structures were designed and divided into two settings based on their number of learnable parameters (i.e., a ~70,000 and a ~290,000 learnable parameter setting). Each setting has three different pooling strategies, i.e., without pooling, with max pooling and with average pooling. Results showed that CNN performed better than the established random forest classifier. This is because the CNN end-to-end training can effectively extract spatial information favorable to discriminate spectrally similar classes which occurs frequently in smallholder agricultural landscapes and especially in the four-band multispectral images. Random forest, despite taking the same 7 × 7 image patch as input, can only consider each pixel separately during the tree split training process.

## Figures and Tables

**Table 1:** Pooling operation, input image patch size and layer number in supervised convolutional neural network (CNN) used in the literature for remote sensing image land cover classification. Only layers with learnable parameters are counted following Simonyan and Zisserman [41] (e.g., pooling operation is not a layer).

Literatures	Pooling	Input Image Patch Size	Layer Number
Chen et al. [42]	max	27 × 27	7
Zhao and Du [43]	max	32 × 32	1, 2, 3, 4, 5
Kussul et al. [29]	max	7 × 7	4
Guo et al. [44]	average	18 × 18	4
Li et al. [45]	no	5 × 5	4
Mei et al. [46]	no	3 × 3; 5 × 5	3
Santara et al. [47]	no	3 × 3	5, 6, 7
Yang et al. [48]	max	21 × 21	4
Wu and Prasad [49]	max	11 × 11	4
Hamida et al. [50]	max	3 × 3; 5 × 5; 7 × 7	5, 7, 9, 11
Ji et al. [31]	average; max	8 × 8	5
Xu et al. [51]	max	7 × 7; 9 × 9; 11 × 11	4
Liu et al. [52]	max	9 × 9	4
Song et al. [53]	average	23 × 23; 25 × 25; 27 × 27	26; 32
Hao et al. [54]	max	7 × 7	5
Zhong et al. [55]	average	3 × 3; 5 × 5; 7 × 7; 9 × 9; 11 × 11	12
Zhang et al. [56]	max	16 × 16	5
Yang et al. [57]	no	7 × 7	5; 10
Mahdianpari et al. [58]	max	30 × 30 and resampled to the input size for each CNN designed in computer vision field	16~152
Gao et al. [59]	max	5 × 5; 7 × 7; 9 × 9	4
Karakizi et al. [60]	max	29 × 29	4
Wei et al. [61]	no	3 × 3	9
Paoletti et al. [62]	average	11 × 11	25
Wang et al. [63]	average	5 × 5; 7 × 7; 9 × 9; 11 × 11; 13 × 13	12
Li et al. [64]	maximum overlap	14 × 14	4

**Table 2:** Characteristics of panchromatic/multispectral sensors aboard on the GaoFen-1 satellite.

Band	Wavelength (nm)	Spatial Resolution (m)	Re-Visiting Period (Days)	Swath (km)
Panchromatic	450–900	2	4	60
Blue	450–520	8		
Green	520–590	8		
Red	630–690	8		
Near infrared	770–890	8		

**Table 3:** Number of training and testing 2 m pixel samples in four study areas. The samples were derived by systematically sampling from the reference land cover maps (Figure 3) and randomly split into training and testing.

Classes	Study Area 1		Study Area 2		Study Area 3		Study Area 4	
	Training	Testing	Training	Testing	Training	Testing	Training	Testing
Maize	28,345	28,345	13,406	13,406	2574	2575	50,067	50,068
Rice	239	239	138,149	138,149	75,530	75,531	5396	5396
Wheat	1878	1879	720	721	none	none	139	139
Soybean	none	none	4458	4458	none	none	411	411
Mung bean	1419	1420	none	none	none	none	none	none
Vegetable	550	550	190	191	1505	1505	380	381
Orchards	none	none	1172	1173	none	none	none	none
Forest	1565	1565	9737	9737	2192	2192	3281	3281
Grassland	415	415	1325	1325	1048	1049	692	693
Wetland	352,942	352,943	none	none	none	none	none	none
Water	none	none	11,033	11,034	2080	2081	2915	2916
Residential	3677	3678	3205	3206	4348	4348	2121	2121
Road	5271	5271	2753	2753	1435	1435	3330	3331
Bare land	957	958	799	800	none	none	1038	1038
Total	397,258	397,263	186,947	186,953	90,712	90,716	69,770	69,775

**Table 4:** Six CNN structures include two settings with significantly different numbers of learnable parameters (~70,000 and ~290,000) each using three pooling strategies. The number of feature map (in the bracket) is different for CNN with and without pooling to guarantee that their total numbers of learnable parameters (*n*) are very similar. Con and FC indicate the convolution and fully connected layer, respectively. The two numbers (with symbol ×) in each cell outside the bracket indicate the convolution kennel size.

	CNN1 with ~70,000 Learnable Parameters			CNN2 with ~290,000 Learnable Parameters		
	No Pooling	Max Pooling	Avg Pooling	No Pooling	Max Pooling	Avg Pooling
*n*	79,504~79,640	77,011~77,255	77,011~77,255	296,208~296,472	290,971~291,455	290,971~291,455
Input	7 × 7 × 4 input reflectance					
Con layer 1	3 × 3 (32)	3 × 3 (59)	3 × 3 (59)	3 × 3 (64)	3 × 3 (119)	3 × 3 (119)
Con layer 2	3 × 3 (32)	3 × 3 (59)	3 × 3 (59)	3 × 3 (64)	3 × 3 (119)	3 × 3 (119)
Con layer 3	3 × 3 (32)	3 × 3 (59)	3 × 3 (59)	3 × 3 (64)	3 × 3 (119)	3 × 3 (119)
FC layer 1	(32)			(64)		
FC layer 2	(8~12: 11 for study area 1, 12 for study area 2, 8 for study area 3 and 11 for study 4)					

**Table 5:** Study areas 1–4 testing samples (Table 3) accuracies of the random forest and the best CNN model (i.e., the CNN with average pooling and ~290,000 learnable parameters). The training sample percent to the total training samples (the value is same for the testing sample in Table 3) is shown in the bracket after class name.

Classes	Random Forest		CNN2-Avg	
	User’s	Producer’s	User’s	Producer’s
**(a)** Study Area 1 Testing Sample (Table 3)				
Maize (7.14%)	89.30	54.84	85.76	85.57
Rice (0.06%)	NaN	0.00	76.25	25.52
Wheat (0.47%)	80.48	17.99	77.56	64.93
Mung bean (0.36%)	82.91	6.83	64.27	44.72
Vegetable (0.14%)	NaN	0.00	41.83	11.64
Forest (0.39%)	89.29	1.60	70.19	52.97
Grassland (0.10%)	NaN	0.00	38.10	1.93
Wetland (88.84%)	93.38	99.57	97.77	98.98
Residential (0.93%)	77.81	44.81	81.25	75.64
Road (1.33%)	79.18	3.68	61.35	39.39
Bare land (0.24%)	98.94	58.46	91.12	82.46
Overall accuracy	93.13		96.09	
Kappa coefficient	0.5586		0.8001	
**(b)** Study Area 2 Testing Sample (Table 3)				
Maize (7.17%)	79.55	35.15	67.99	62.77
Rice (73.90%)	87.17	97.30	92.98	95.62
Wheat (0.39%)	75.00	1.25	44.38	30.65
Soybean (2.38%)	82.19	67.16	80.88	78.49
Vegetable (0.10%)	NaN	0.00	46.38	16.75
Orchards (0.63%)	95.97	10.14	72.71	55.41
Forest (5.21%)	58.76	57.76	72.32	72.76
Grassland (0.71%)	NaN	0.00	18.39	8.30
Water (5.90%)	76.92	64.40	77.90	74.71
Residential (1.71%)	62.15	61.67	69.37	65.35
Road (1.47%)	71.92	27.82	62.53	51.22
Bare land (0.43%)	100.00	0.13	28.57	17.00
Overall accuracy	84.47		87.72	
Kappa coefficient	0.5939		0.7121	
**(c)** Study Area 3 Testing Sample (Table 3)				
Maize (2.84%)	89.71	4.74	76.84	34.80
Rice (83.26%)	91.55	99.38	94.59	98.62
Vegetable (1.66%)	66.40	10.90	58.62	48.57
Forest (2.42%)	52.25	20.67	61.00	53.01
Grassland (1.16%)	25.00	0.10	34.41	13.25
Water (2.29%)	69.70	58.91	73.03	67.42
Residential (4.79%)	64.94	80.13	79.15	77.90
Road (1.58%)	89.01	22.02	65.21	47.67
Overall accuracy	88.96		91.37	
Kappa coefficient	0.5463		0.6842	
**(d)** Study Area 4 Testing Sample (Table 3)				
Maize (71.76%)	94.11	97.73	96.03	97.38
Rice (7.73%)	89.36	87.82	90.96	92.90
Wheat (0.20%)	87.10	38.85	70.63	81.30
Soybean (0.59%)	88.40	63.02	92.57	84.91
Vegetable (0.54%)	NaN	0.00	17.98	8.40
Forest (4.70%)	51.57	64.68	68.56	68.12
Grassland (0.99%)	86.36	5.48	35.18	21.07
Water (4.18%)	94.89	80.86	93.95	92.15
Residential (3.04%)	62.91	87.32	70.98	75.53
Road (4.77%)	52.20	33.86	61.69	56.32
Bare land (1.49%)	38.01	13.58	37.30	33.82
Overall accuracy	88.53		90.52	
Kappa coefficient	0.7464		0.7959	

**Table 6:** Balanced sample accuracies of the random forest and the CNN2-Avg model for study areas 1–4 samples data. A total of 2000 training and 2000 testing samples were randomly selected from Table 3 (class with samples <4000 was not used). The training sample percent to the total training samples (the value is same for the testing sample) is shown in the bracket after class name.

Classes	Random Forest		CNN2-Avg	
	User’s	Producer’s	User’s	Producer’s
**(a)** Study Area 1 Testing Sample				
Maize (25.00%)	84.38	82.95	85.62	88.40
Wetland (25.00%)	76.74	72.60	81.88	79.30
Residential (25.00%)	83.21	86.00	89.66	87.10
Road (25.00%)	64.92	67.35	72.80	74.80
Overall accuracy	77.26		82.40	
Kappa coefficient	0.6963		0.7653	
**(b)** Study Area 2 Testing Sample				
Maize (14.29%)	74.92	57.35	71.18	65.95
Rice (14.29%)	69.43	80.05	74.50	79.45
Soybean (14.29%)	83.25	89.20	88.73	90.90
Forest (14.29%)	60.66	70.85	73.16	73.85
Water (14.29%)	82.09	62.35	73.89	74.15
Residential (14.29%)	75.58	79.70	85.44	85.10
Road (14.29%)	63.23	65.00	77.25	75.20
Overall accuracy	70.73		77.80	
Kappa coefficient	0.6742		0.7410	
**(c)** Study Area 3 Testing Sample				
Maize (20.00%)	76.43	67.95	80.57	79.00
Rice (20.00%)	65.60	72.75	73.49	76.50
Forest (20.00%)	58.58	67.45	73.27	75.10
Water (20.00%)	82.36	69.80	83.14	80.60
Residential (20.00%)	78.61	78.85	86.28	84.90
Overall accuracy	70.08		79.22	
Kappa coefficient	0.6420		0.7403	
**(d)** Study Area 4 Testing Sample				
Maize (16.67%)	92.19	86.70	89.74	89.70
Rice (16.67%)	93.00	89.65	91.78	92.70
Forest (16.67%)	61.89	74.80	74.81	72.75
Water (16.67%)	96.06	81.70	93.47	91.60
Residential (16.67%)	83.88	92.65	86.85	89.50
Road (16.67%)	66.47	61.95	72.85	73.40
Overall accuracy	81.15		84.94	
Kappa coefficient	0.7749		0.8193	
